# Misdiagnosed with spinal infection instead of SAPHO syndrome: a case report and literature review

**DOI:** 10.3389/fimmu.2025.1683214

**Published:** 2025-10-16

**Authors:** Shuai Wang, Shiyong Wan, Zhengqi Chang

**Affiliations:** ^1^ Department of Orthopedics, 960th Hospital of People's Liberation Army, Jinan, China; ^2^ Department of Orthopedics, 961th Hospital of People's Liberation Army, Qiqihaer, China

**Keywords:** SAPHO syndrome, spinal infection, misdiagnosis, imaging, case report

## Abstract

This article reports a case of SAPHO syndrome misdiagnosed as spinal infection, detailing the clinical manifestations, laboratory tests, and imaging features of the patient. Through a review of the literature, common confounding factors and reasons for misdiagnosis in the diagnostic process of this disease are summarized. Comprehensive analysis of multiple related studies reveals that detailed medical history collection, advanced imaging examinations (such as ECT), and multidisciplinary consultations play a decisive role in making a correct diagnosis. Furthermore, the literature shows that the treatment for SAPHO syndrome is fundamentally different from spinal infection, with the former mainly relying on anti-inflammatory regulation, bone protective agents, and immune modulation therapy, rather than the indiscriminate use of antibiotics. This report aims to remind clinicians to maintain a high level of vigilance when facing spinal lesions, and to compare and summarize the latest advances in diagnosis and treatment, providing a reference for improving the level of diagnosis and treatment in the future.

## Introduction

SAPHO syndrome is a rare autoimmune inflammatory disease, named after the initials of Synovitis, Acne, Pustulosis, Hyperostosis, and Osteitis. It presents with a variety of clinical manifestations, often posing a diagnostic challenge. The incidence rate of SAPHO syndrome is less than 1/10,000. It predominantly affects adults, with a female predominance (1). The disease can manifest as skin problems (such as acne, palmoplantar pustulosis) and can also involve the skeletal system, particularly osteitis and hyperostosis, with spinal lesions receiving increasing attention (2). Due to similarities in imaging and some laboratory indicators, patients are often initially misdiagnosed with chronic infectious spondylitis or other bacterial infectious diseases, leading to unnecessary antibiotic treatment and potential surgical interventions (3, 4).

Recent studies have reported an increasing number of cases of spinal involvement in SAPHO syndrome. Spinal involvement often presents with inflammation and bone changes at the vertebral edges, intervertebral discs, and adjacent soft tissues, with imaging showing both bone destruction and bone proliferation, easily confused with infectious spondylitis (5, 6). Additionally, research indicates that bone inflammation in SAPHO syndrome is often a “reactive bone inflammation,” not caused by direct infection, fundamentally distinguishing it from true infectious cases (7).

This report aims to analyze the diagnostic challenges and reasons for misdiagnosis of SAPHO syndrome when the spine is involved, as well as discuss appropriate diagnostic procedures and targeted treatment strategies. It provides clinicians with more differential diagnosis information to reduce the risk of misdiagnosis (4, 8).

## Case present

**Figure 1:**
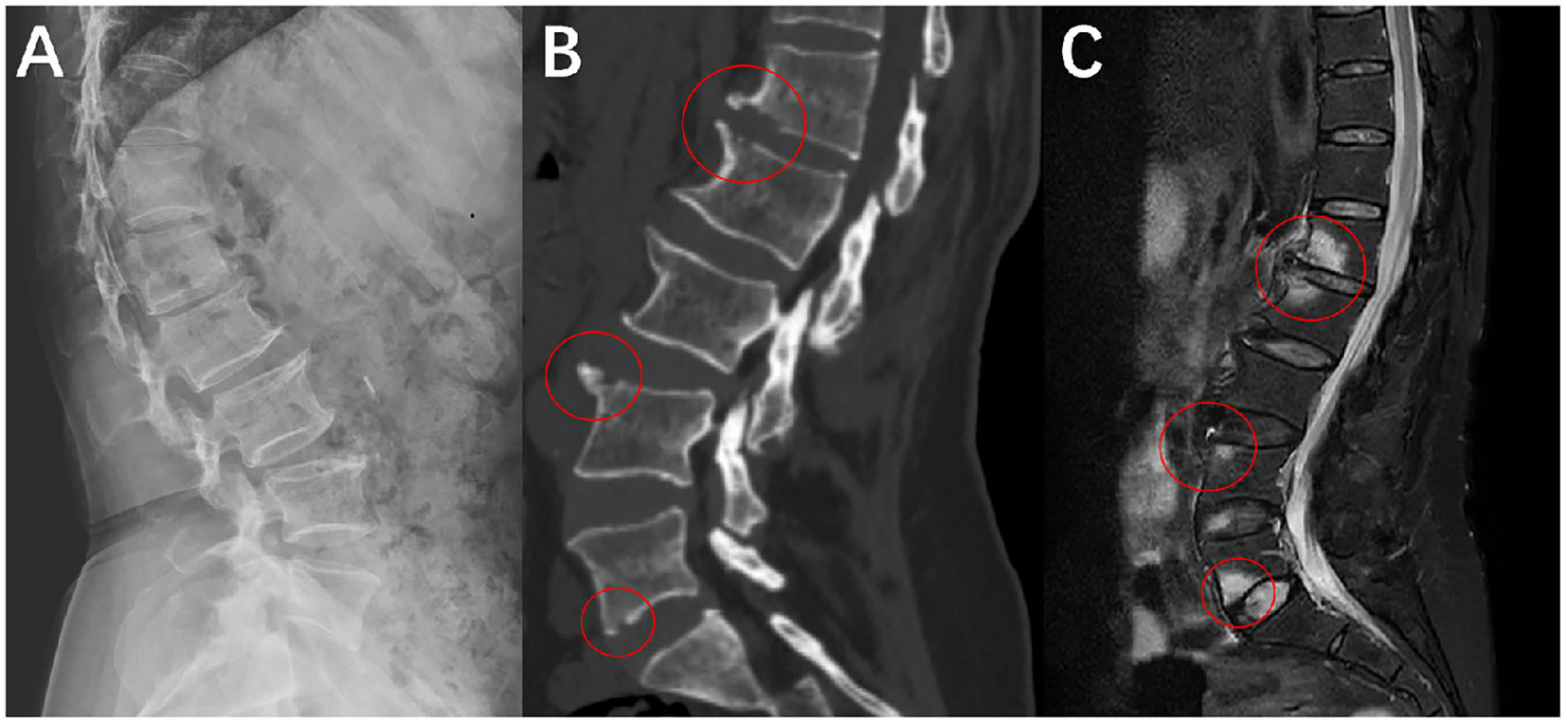
The image shows a mixed change of osteogenesis and bone destruction. **(A)** X-ray examination, shows bone hyperplasia at the anterior edge of the vertebral body in the lateral view of the lumbar spine. **(B)** CT examination, and the position within the red circle can more clearly demonstrate the coexistence of bone hyperplasia and bone destruction. **(C)** MRI examination, and the red circle indicates vertebral edema and local bone marrow reaction.

The patient is a 59-year-old female presenting with a 1-month history of persistent low back pain. She had no significant medical history, including trauma or chronic illnesses. Initial evaluation at a local orthopedic clinic revealed recurrent fever, nighttime pain awakening, and localized tenderness. Serial blood tests demonstrated elevated inflammatory markers (ESR: 81 mm/h, CRP: 15.58 mg/L on admission; ESR: 43 mm/h, CRP: 13.27 mg/L at 1-week follow-up), though blood cultures remained negative for pathogens. At the initial admission, an echocardiogram showed no abnormal changes suggestive of infective endocarditis or other conditions, while Magnetic resonance imaging (MRI) indicated chronic spinal infection with vertebral edema (3) (**Figure 1**).

**Figure 2:**
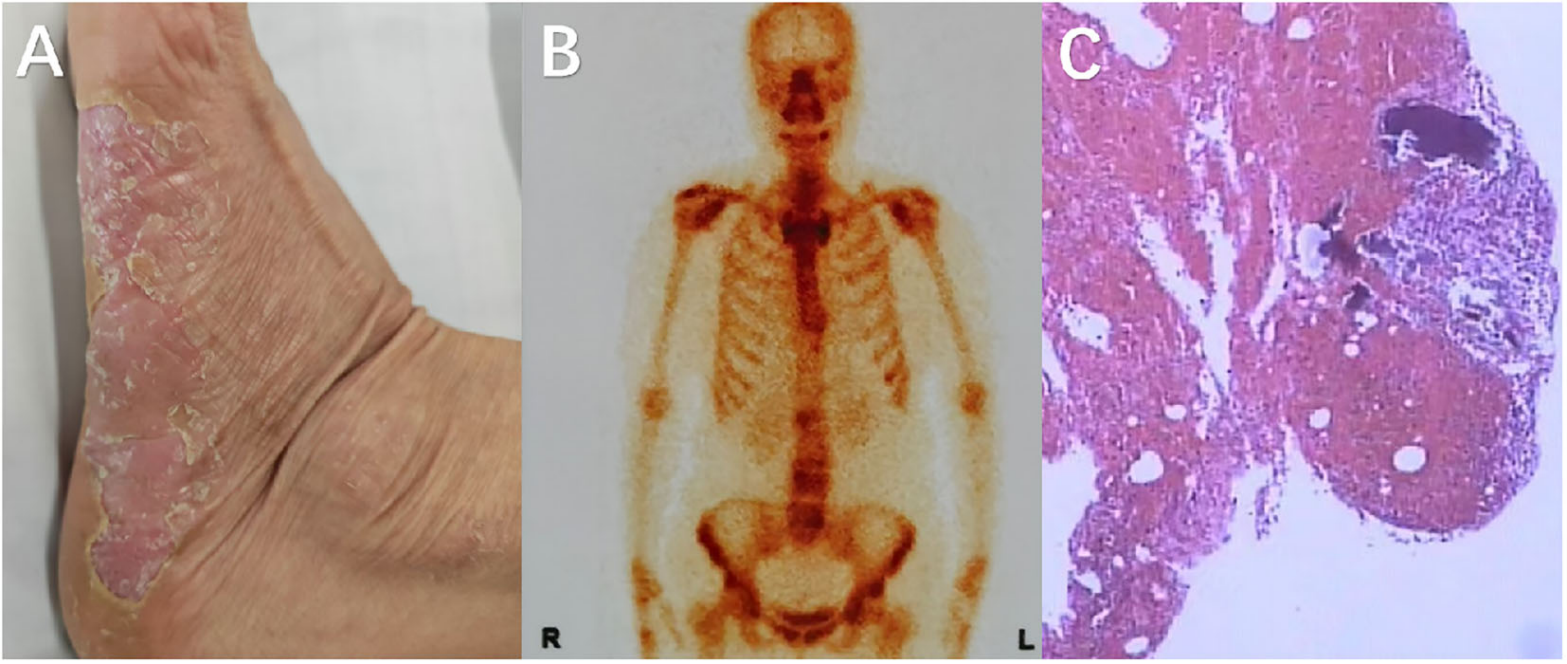
**(A)** Pustular changes on the plantar surface of the left foot; **(B)** ECT examination reveals the coexistence of local bone hyperplasia and bone destruction in some areas of the patient’s spine, with multiple lesions distributed irregularly (L1, L2, L4, L5); **(C)** The pathology shows inflammatory cell infiltration, mainly lymphocytes and plasma cells, with occasional neutrophils, but no evidence of pathogens.

Initial management involved a 4-week course of broad-spectrum antibiotics based on imaging findings and inflammatory markers, yet clinical improvement was absent. The patient was then transferred to our department for further treatment. On physical examination: pustular skin lesions were observed on both hands and feet; the range of motion in lumbar flexion and extension was limited; extensive tenderness and percussion pain were present over the lumbodorsal region; bilateral Patrick’s test (Fabere test) was negative; bilateral straight leg raise test of the lower extremities was negative; bilateral patellar tendon reflexes and Achilles tendon reflexes were normal; and all pathological signs were negative. Emission Computed Tomography (ECT) showed increased radionuclide uptake in the bilateral first sternocostal joints, manifested as the ““bull’s head sign”, as well as multifocal vertebral lesions(L1, L2, L4, L5). These vertebral lesions showed the coexistence of bone sclerosis and osteolytic destruction, which was inconsistent with typical infectious spondylitis (9). Considering that the patient might not have spinal infection, a multidisciplinary consultation was initiated for evaluation. The dermatology department consultation confirmed the diagnosis of palmoplantar pustulosis, and subsequently, the rheumatology and immunology department consultation suggested a high possibility of SAPHO syndrome. A CT-guided biopsy of the L1 vertebra under local anesthesia demonstrated neutrophilic and plasma cell infiltration. Immunohistochemical staining results: CD138(+), Lambda(+), MP0 (focal+), P53 (scattered+), CD3 (partial+), CD56 (focal+), CD79a (partial+), CD20 (scattered+), Kappa (scattered+), IgG4(-), CK(-), SATB2(-), Ki-67 (~80%+). Special stains: PAS(-) (**Figure 2**).

The constellation of clinical features (notably palmoplantar pustulosis), imaging abnormalities, and histopathology confirmed SAPHO syndrome (3). Discontinuation of antibiotics and initiation of NSAIDs, immunosuppressive therapy, and anti-osteoporotic agents led to marked symptomatic improvement within 3 months. Follow-up laboratory tests showed normalization of inflammatory markers, and repeat imaging demonstrated stable lesions without progression.

## Discussion

The issue of misdiagnosis in SAPHO syndrome has long perplexed clinicians. In our case, the patient initially presented with chronic back pain and abnormal local inflammatory markers. MRI revealed extensive involvement of the spine, demonstrating both inflammatory lesions and bone repair, which led the clinical team to favor an infectious etiology. For instance, the MRI findings of vertebral edema and localized bone marrow reactions in this case were initially misinterpreted as chronic spondylodiscitis. However, the lack of response to antibiotics and concurrent skin lesions were overlooked as critical clues (3). Imaging plays a pivotal role in confirming SAPHO syndrome. While conventional MRI is sensitive to inflammatory changes, it lacks specificity for detecting bone-interface abnormalities. Modalities such as CT and ECT better delineate the coexistence of hyperostosis and osteolysis, which aids in distinguishing SAPHO from infectious spinal lesions (9). Deng et al. further validated this observation, demonstrating that SAPHO-related spinal lesions exhibit mixed imaging features distinct from purely infectious foci (5).

Laboratory findings, though suggestive, are insufficient for definitive diagnosis. As highlighted by Huang et al., many SAPHO patients exhibit elevated inflammatory markers despite negative microbiological studies, underscoring the need to recognize “pseudo-infectious” presentations (7). Biopsy and histopathology are diagnostic: sterile inflammation dominated by lymphocyte and plasma cell infiltration (rare neutrophils), absence of pathogens, hyperostosis with periosteal reaction and new bone formation, marrow fibrosis with chronic inflammation, and lack of necrosis or granulomas—features inconsistent with infection.

Another contributor to misdiagnosis is insufficient clinician awareness. Early literature framed SAPHO as rare, leading to its exclusion in routine spinal evaluations. Recent case reports and systematic reviews, however, suggest its prevalence is underestimated. Notably, when the patient was treated at a local orthopedic clinic, palmoplantar pustular skin lesions had already developed; however, the clinicians did not conduct a thorough physical examination nor consult the dermatology department for further evaluation. Therefore, caution should be exercised when making a diagnosis relying solely on imaging examinations and blood test results, without adequate medical history taking and physical examination. Multidisciplinary collaboration (e.g., dermatology, rheumatology, immunology, radiology) significantly improves diagnostic accuracy (8, 10).

Treatment strategies differ starkly between SAPHO and spinal infections. Prolonged antibiotics or surgery are mainstays for infections, whereas SAPHO requires anti-inflammatory agents, immunomodulatory therapy, and bone-protective strategies. An expert consensus in 2025 named SAPHO syndrome as chronic non-bacterial osteitis(CNO) in adults and proposed a phased treatment plan: first-line treatment uses non-steroidal anti-inflammatory drugs (NSAIDs) or cyclooxygenase-2 inhibitors (COXIBs); second-line treatment requires adding or switching to intravenous bisphosphonates (IVBP, generally preferred) or tumor necrosis factor-α inhibitors (TNFi); third-line treatment requires referral to a specialist center to obtain a third-line treatment plan (11). Misdiagnosis often results in futile antibiotic regimens, delaying effective treatment, increasing adverse events, and escalating healthcare burdens (12). Early recognition is thus critical.

In summary, this case and literature review emphasize that spinal SAPHO is easily confused with chronic spinal infection. Key lessons include: integrating clinical history (e.g., cutaneous manifestations), multimodal imaging, laboratory data, and histopathology; and advocating multidisciplinary evaluation to optimize diagnosis and management.

## Data Availability

The original contributions presented in the study are included in the article/supplementary material. Further inquiries can be directed to the corresponding author.
